# Neurotrophic effects of neudesin in the central nervous system

**DOI:** 10.3389/fnins.2013.00111

**Published:** 2013-06-25

**Authors:** Ikuo Kimura, Yoshiaki Nakayama, Ying Zhao, Morichika Konishi, Nobuyuki Itoh

**Affiliations:** ^1^Department of Pharmacogenomics, Kyoto University Graduate School of Pharmaceutical ScienceKyoto, Japan; ^2^Laboratory of Neuroglycobiology, Department of Molecular Sciences, Faculty of Life Sciences, Kyoto Sangyo UniversityKyoto, Japan; ^3^Department of Microbial Chemistry, Kobe Pharmaceutical UniversityKobe, Japan; ^4^Department of Genetic Biochemistry, Kyoto University Graduate School of Pharmaceutical ScienceKyoto, Japan

**Keywords:** MAPR, heme-binding protein, PGRMC1, neudesin, NENF, progesterone

## Abstract

Neudesin (neuron-derived neurotrophic factor; NENF) was identified as a neurotrophic factor that is involved in neuronal differentiation and survival. It is abundantly expressed in the central nervous system, and its neurotrophic activity is exerted via the mitogen-activated protein kinase (MAPK) and phosphatidylinositol 3-kinase (PI3K) pathways. Neudesin is also an anorexigenic factor that suppresses food intake in the hypothalamus. It is a member of the membrane-associated progesterone receptor (MAPR) family and shares key structural motifs with the cytochrome b5-like heme/steroid-binding domain. Progesterone receptor membrane component 1 (PGRMC1), the first to be discovered among the MAPR family, binds progesterone to induce “rapid non-genomic effects” in biological responses that are unrelated to the nuclear progesterone receptors (PRs). Hence, neudesin may also be involved in the rapid non-genomic actions of progesterone. In this review, we summarize the identification, structure, and activity of neudesin in the central nervous system, and present an essential overview of the current understanding of its physiological roles and the prospect of elucidating its non-genomic progesterone effects.

## Introduction

Steroid hormones, such as estrogen and progesterone, are known to exert their physiological effects via their specific nuclear receptors (O'Malley and Means, 1974). Steroid hormones modulate gene transcription by interacting with these nuclear receptors, which act as ligand–dependent transcription factors. These effects are called the “genomic” actions of steroid hormones, which generally take a few hours to days to manifest fully. However, in some tissues, including the central nervous system, steroid hormones have been found to act rapidly (within minutes,) on the targeted cells, independent of their nuclear receptor, suggesting the possible involvement of unidentified receptors in the rapid non-genomic actions of steroid hormones (Falkenstein and Wehling, 2000). However, the putative receptors for these actions have not been identified.

**Figure 1:**
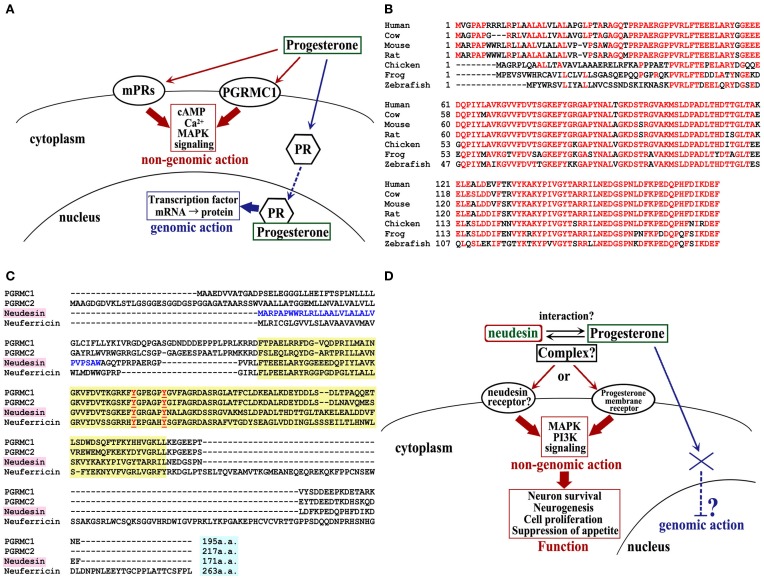
**MAPR family signaling, neudesin homolog in various species, and multiple sequence alignment of mouse MAPR family. (A)** Membrane progesterone receptor signaling. PR, nuclear progesterone receptor. **(B)** Neudesin orthologs of human (NM_013349), cow (NM_001076419), mouse (NM_025424), rat (NM_001002851), chicken (XM_419430), frog (NM_001030454), and zebrafish (NM_001037704). The consensus sequence is shown in red. **(C)** The cytochrome b5 heme-binding (Cyt-b5) domain is indicated by the yellow box. Two conserved tyrosine residues, which are important for heme binding, are shown in red. The predicted signal peptides of neudesin are shown in blue. **(D)** Putative neudesin signaling.

During and after the 1990s, GPR30, one of the typical G-protein coupled receptors (GPCRs), and membrane progesterone receptors (mPRs), which are putative GPCRs, were identified as the membrane receptors for estrogen and progesterone, respectively (Thomas, 2008; Thomas et al., 2009; Prossnitz and Barton, 2011). Moreover, progesterone receptor-membrane component (PGRMC)1 and PGRMC2, 2 single transmembrane proteins, were also identified as putative membrane receptors for progesterone (Meyer et al., 1996; Cahill, 2007; Peluso, 2007). In contrast to the nuclear receptors, these membrane receptors mediate rapid non-genomic effects of the steroid hormones, such as the activation of mitogen-activated protein kinase (MAPK) signaling and intracellular Ca^2+^ increases (Thomas, 2008; Zhu et al., 2008; Kimura et al., 2012; Figure 1A).

Recently, we identified neudesin (neuron-derived neurotrophic secreted protein) and neuferricin, (neuron-derived extracellular heme-binding protein, which have high homology to PGRMC1 and PGRMC2 (Kimura et al., 2005, 2010). These four proteins (i.e., PGRMC1/2, neudesin, and neuferricin) belong to the membrane-associated progesterone receptor (MAPR) family (Kimura et al., 2012). However, PGRMC1 and PGRMC2 are located on the cell membrane, whereas neudesin and neuferricin are secreted proteins. Neudesin potentially promotes neuronal differentiation and survival *in vitro* by activating the MAPK and phosphatidylinositol-3-kinase (PI-3K) signaling pathways (Kimura et al., 2005, 2006). These effects on cultured neural cells, together with its expression profiles, suggest that neudesin may play crucial roles in the development and maintenance of the central nervous system. Interestingly, neudesin is also reported to bind to heme by a cytochrome b5-like heme/steroid-binding domain that is conserved among the MAPR family, and this binding is essential for the activities of neudesin *in vitro* (Kimura et al., 2008). However, the specific receptor for neudesin and the functional significance of the heme–neudesin interaction have not yet been clarified.

Since, in various tissues, PGRMC1 is involved in the rapid non-genomic effects of progesterone, it is expected that neudesin may also regulate the rapid effects of progesterone. However, to date, there are no reports on the rapid non-genomic effects of steroid hormones regulated by neudesin. Here, we give an overview of neudesin, including its identification, expression, and structural features. In addition, its physiological roles, possible involvement in progesterone signaling, and future prospects are discussed.

## Structure

Neudesin cDNAs have been isolated by PCR from embryonic day-18 mice and from the human brain by PCR (Kimura et al., 2005).

Mouse neudesin cDNA encodes a secretory 171-amino-acid protein, with a putative signal sequence at its N-terminus. Its homolog exists in various species, including fish, amphibians, birds, and vertebrates from mice to humans. To date, the neudesin gene has not yet been found in invertebrates, including *C. elegans, D. melanogaster*, and *C. intestinalis*. The N-terminal differs among mammals and other species, but except for this region, high amino acid homology exists (Figure 1B). Neudesin contains a heme/steroid-binding domain that is similar to those of PGRMC1 and cytochrome b5 (Cyt-b5) (Kimura et al., 2008). As mentioned above, neudesin belongs to the MAPR family, which composed of PGRMC1, PGRMC2, neudesin, and neuferricin, and is a subfamily of the Cyt-b5 family, which consists of heme-binding proteins with a Cyt-b5-like heme/steroid-binding domain in their central regions (Kimura et al., 2012; Figure 1C).

The structure of human neudesin has been obtained by NMR; it involves a β1-α1-β2-β3-α 2-β4-α 3-α 4-β 5-β 6 structure that contains 4 α-helices and 6 β-strands, and the heme-binding pocket is predicted to be formed between α 2 and α 3 (Han et al., 2012). The sequence from residues 45–143 in mouse neudesin is a putative heme-binding domain, in which Tyr-81 and Tyr-87 form the predicted binding site for the heme iron (Figure 1C). Hence, recombinant mouse neudesin can bind to heme, whereas a mutant recombinant mouse neudesin that lacks this putative heme-binding domain cannot bind to heme (Kimura et al., 2008).

## Expression

In mouse embryos, neudesin is expressed in several regions; in particular, it is expressed most abundantly in the developing brain and spinal cord (Kimura et al., 2005). In the embryonic cerebral cortex, neudesin is expressed mainly in the preplate, which consists of post-mitotic neural cells after migration, but not in the subventricular zone and ventricular zone, which consist mainly of neural progenitor cell/stem cells that are replication competent (Kimura et al., 2006). Furthermore, *in vitro*, the expression of neudesin is gradually reduced after the induction of neurogenesis in neural progenitor cells; thereafter, it is hardly expressed in the early stages of differentiated neurons.

In contrast to the expression pattern in the embryo, neudesin is abundantly expressed in various tissues—in the adult mouse, including the brain, heart, lung, and kidney. In the mouse brain, neudesin is expressed in neurons of every brain region, in particular in the cerebral cortex, hippocampus, thalamus, and hypothalamus, but it is not expressed in glial cells (Kimura et al., 2005). These differences in neudesin expression between embryonic-stage and adult-stage neural cells suggest that neudesin may play distinct roles in progenitor neural cells and in mature neurons.

## Signal transduction

Interestingly, neudesin signal transduction differs among neural cell types in the central nervous system. The neurotrophic effects of neudesin in primary cultured neurons are exerted via the MAPK and PI-3K pathways. In addition, pertussis toxin (PTX), a G_i_/G_o_ protein inhibitor, significantly inhibits the phosphorylation of extracellular signal-regulated kinase (ERK)1/2 by neudesin, indicating that the neurotrophic activity of neudesin may be related to the activation of a G_i_/G_o_ protein-coupled receptor (Kimura et al., 2005). On the other hand, neudesin also promotes the phosphorylation of ERK, Akt, and cAMP response element binding protein (CREB) in neural precursor cells; however, the effect of neudesin is not inhibited by PTX, unlike the case in primary neurons. In addition, since neudesin increases cAMP levels in neural precursor cells, the G_s_ protein-coupled receptor may be involved in the activation of the MAPK, protein kinase A (PKA), and PI-3K signaling pathways by neudesin (Kimura et al., 2006). To date, our group and others have reported that neudesin activates the MAPK and PI-3K signaling pathways in cancer cells and in other tissues, besides the nervous system, such as adipose tissues (Kimura et al., 2009; Han et al., 2012). However, there is no evidence that neudesin binds directly to these GPCRs of the G-protein type. Moreover, although neudesin is an extracellular protein, no specific receptor(s) for neudesin has been reported to date. Hence, the identification of the neudesin receptor(s), or proteins that interact directly with neudesin, may yield insight into the neudesin signal transduction system and neudesin function.

## Functions

With regard to the physiological functions of neudesin in the central nervous system, to date, it has been reported that neudesin acts as a neurotrophic factor in postnatal mature neurons, enhancing neuronal survival (Kimura et al., 2005), and promotes cell proliferation and neurogenesis in undifferentiated neural progenitor cells at the embryonic stage (Kimura et al., 2006). In mouse primary cultured cerebral cortex neurons, recombinant neudesin significantly increases neuronal survival by decreasing apoptosis, whereas neudesin exhibited no cell proliferation activity in mouse primary cultured cerebral cortex astrocytes (Kimura et al., 2005). This indicates that neudesin, secreted from neurons, acts on these or neighboring neurons, but not on astrocytes. During brain development, recombinant neudesin increases neuronal numbers directly by inducing the differentiation of neural precursor cells into microtubule-associated protein 2 (MAP-2)-positive neurons through the PI-3K and PKA pathways, and indirectly by transiently promoting the proliferation of the dividing neural precursor cells through the MAPK and PKA pathways, but not via the PI-3K pathway (Kimura et al., 2006). Similarly, in Neuro2a cells (mouse neuroblastoma cells), an undifferentiated neural cell line, cell survival, and proliferation were significantly suppressed by siRNA-mediated knockdown of endogenous neudesin (Kimura et al., 2008). Moreover, interestingly, a neudesin–hemin complex promoted stronger neuroprotective activity in primary cultured neurons than did neudesin alone; similarly, the neudesin–hemin complex, but not neudesin or hemin alone, induced survival and proliferation in Neuro2a cells (Kimura et al., 2008). These results indicate that heme binding to neudesin is essential for the bioactivity of neudesin. However, it is still not clear by which mechanism heme contributes to the effects of neudesin. It is possible that the charge of the heme iron is involved in the binding of neudesin to its receptor, or in the interactions between neudesin and other proteins. Very recently, neudesin in the hypothalamus has been reported to modulate appetite (Byerly et al., 2013). Hypothalamic neudesin mRNA was regulated by brain-derived neurotrophic factor (BDNF) signaling, an important regulator of appetite. Moreover, recombinant neudesin that was administered via an interacerebroventricular cannula decreased food intake and body weight through activation of melanocortin signaling, by increasing the hypothalamic Pomc and Mc4r mRNA expression. This is the first report of a physiological function for neudesin *in vivo*.

As described above, the secretory heme-binding protein, neudesin, is highly expressed in the central nervous system, and functions as a neurotrophic factor to regulate neuronal differentiation and survival by activating the MAPK and PI-3K pathways *in vitro*, and as an anorexigenic factor in the hypothalamus *in vivo* (Table 1). It is possible that neudesin also plays important physiological roles in the central nervous system *in vivo*. The analysis of the knockout mice will further elucidate its physiological functions.

**Table 1:** **Functions of neudesin**.

**Neural cell type or brain area**	**Function**	**Signal transduction**	**Experimental method**
Mouse neurons	Cell survival	MAPK and PI3K	Addition of recombinant protein
Mouse neural precursor cells	Promotion of neurogenesis transient cell proliferation	PI3K	Addition of recombinant protein
		MAPK	
Neuro2a cells (mouse neuroblastoma)	Cell survival	MAPK and PI3K	Addition of recombinant protein RNA interference
	Cell proliferation		
Mouse hypothalamus	Suppression of appetite	−	Administration of recombinant protein via i.c.v

## Does neudesin interact with the progesterone-signaling pathway?

To date, there is no evidence to support the interaction between neudesin and progesterone, other than its structural similarity to PGRMC1, which binds to progesterone with a similar affinity as it does to other steroids (Meyer et al., 1996). However, previous case studies of human diseases provide some possible answers to this question. One case report demonstrated that expression levels of the progesterone receptor (PR) in estrogen receptor (ER)-positive breast cancer cells correlated positively with the responsiveness to tamoxifen, and with the prognosis (Fisher et al., 2001). Intriguingly, neudesin protein was also upregulated in ER^+^/PR^+^ tumors, compared with ER^+^/PR^−^ tumors (Neubauer et al., 2006). These reports raise the possibility that neudesin also interacts with progesterone, and is associated with the pharmacological effect of tamoxifen.

In addition to the fact that the PGRMC1 complex binds to progesterone, Liu et al. showed that the growth effect of progesterone on neural precursor cells is mediated by membrane-associated PGRMC1 and PGRMC2, via activation of the MAPK pathway, as in the case of neudesin (Kimura et al., 2006; Liu et al., 2009). Furthermore, progesterone was reported to directly and rapidly inhibit GnRH neuronal activity via PGRMC1 (Bashour and Wray, 2012). These reports suggest that PGRMCs are membrane receptors for progesterone, and are involved in immediate-type reactions to progesterone. Moreover, it has been reported that progesterone binds directly to PGRMC1 (Peluso et al., 2008). On the other hand, other reports suggest that PGRMC1 is indirectly related to progesterone activity because of adrenal steroidogenesis, but it does not bind directly to progesterone (Min et al., 2005). Nevertheless, PGRMC1 is closely involved in cellular responses to progesterone. If future research reveals an interaction between neudesin and progesterone, this could provide the missing link regarding to the progesterone activity.

Interestingly, neudesin is a secretory protein, unlike PGRMC1. Since progesterone is lipophilic, the consensus model for the mechanism of progesterone activity holds that the steroid hormone penetrates the plasma membrane and gains access to classical nuclear PRs in order to exert its biological functions. However, it is assumed that the “rapid effects” of progesterone depend upon interactions with certain cell surface molecules. It is then not clear why such a lipophilic molecule is trapped on the cell surface, without penetrating the plasma membrane into the cytoplasm, and whether this occurs accidentally. It is possible that progesterone binds to proteins that negate its liposolubility, thus overcoming this problem. Neudesin may function as such a progesterone-binding protein in the extracellular environment. Formation of a progesterone–neudesin complex may suppress the transference of progesterone to its nuclear receptor; alternatively, the complex may act on a cell surface protein, such as an unknown PR (Figure 1D). The elucidation of the novel mechanisms regulating the neudesin-mediated progesterone effects will present a breakthrough in sex steroid hormone research in the future.

### Conflict of interest statement

The authors declare that the research was conducted in the absence of any commercial or financial relationships that could be construed as a potential conflict of interest.
